# Endogenous C-type natriuretic peptide offsets the pathogenesis of steatohepatitis, hepatic fibrosis, and portal hypertension

**DOI:** 10.1093/pnasnexus/pgae579

**Published:** 2024-12-30

**Authors:** Cristina Perez-Ternero, Wenhao Li, Aisah A Aubdool, Robert D Goldin, John Loy, Kalpana Devalia, William Alazawi, Adrian J Hobbs

**Affiliations:** Faculty of Medicine and Dentistry, William Harvey Research Institute, Barts and The London, Queen Mary University of London, Charterhouse Square, London EC1M 6BQ, United Kingdom; Barts Liver Centre, Blizard Institute, Queen Mary University of London, 4 Newark Street, London E1 2AT, United Kingdom; Faculty of Medicine and Dentistry, William Harvey Research Institute, Barts and The London, Queen Mary University of London, Charterhouse Square, London EC1M 6BQ, United Kingdom; Centre for Pathology, St Mary’s Hospital, Imperial College, London W2 1NY, United Kingdom; Bariatric Surgery Department, Homerton University Hospital, Homerton Row, London E9 6SR, United Kingdom; Bariatric Surgery Department, Homerton University Hospital, Homerton Row, London E9 6SR, United Kingdom; Barts Liver Centre, Blizard Institute, Queen Mary University of London, 4 Newark Street, London E1 2AT, United Kingdom; Faculty of Medicine and Dentistry, William Harvey Research Institute, Barts and The London, Queen Mary University of London, Charterhouse Square, London EC1M 6BQ, United Kingdom

**Keywords:** natriuretic peptide receptor, G-protein-coupled receptor, metabolic dysfunction-associated steatotic liver disease, portal hypertension, fibrosis

## Abstract

Metabolic dysfunction-associated steatotic liver disease (MASLD), hepatic fibrosis, and portal hypertension constitute an increasing public health problem due to the growing prevalence of obesity and diabetes. C-type natriuretic peptide (CNP) is an endogenous regulator of cardiovascular homeostasis, immune cell reactivity, and fibrotic disease. Thus, we investigated a role for CNP in the pathogenesis of MASLD. Wild-type (WT), global CNP (gbCNP^−/−^), and natriuretic peptide receptor-C (NPR-C^−/−^) knockout mice were fed a choline-deficient defined amino acid diet or administered CCl_4_. Liver damage was assessed by histological and biochemical analyses, with steatosis and portal vein size determined by ultrasound. Portal vein pressure and reactivity were measured in vivo and ex vivo, respectively. Pharmacological CNP delivery was used to evaluate prospective therapeutic benefit, and plasma CNP concentration was compared in controls and patients with cirrhosis. Circulating CNP concentration was lower in patients with cirrhosis compared with controls. gbCNP^−/−^ mice were more susceptible, versus WT, to advanced steatohepatitis and hepatic fibrosis, characterized by increased immune cell infiltration, fibrosis, ballooning, plasma alanine aminotransferase concentration, and up-regulation of markers driving these processes. gbCNP^−/−^ mice had increased portal vein diameter and pressure, underpinned by CNP insensitivity. NPR-C^−/−^ animals recapitulated, comparatively, the exaggerated pathogenic phenotype in gbCNP^−/−^ mice, whereas CNP reduced hepatic stellate cell proliferation via NPR-B-dependent inhibition of extracellular signal-related kinase 1/2. Administration of CNP reversed many aspects of disease severity. These data define a new intrinsic role for CNP in offsetting the pathogenesis of MASLD, hepatic fibrosis, and portal hypertension and the potential for targeting CNP signaling for treating these disorders.

**Significance Statement**

Metabolic dysfunction-associated steatotic liver disease (MASLD) and its complications, particularly fibrosis and portal hypertension, constitute an increasing public health problem due to the growing prevalence of obesity and diabetes. Herein, we establish that intrinsic production of C-type natriuretic peptide (CNP) prevents and reverses the development of steatohepatitis and is particularly effective in reducing portal hypertension. Thus, pharmacological targeting of CNP represents a new therapeutic opportunity in MASLD and portal hypertension.

## Introduction

Metabolic dysfunction-associated steatotic liver disease (MASLD), defined as an excessive accumulation of fat in the liver in the presence of a metabolic risk factor (1, 2), is estimated to affect more than 30% of the Western population (3) in keeping with rising rates of obesity and diabetes mellitus type 2 (4). In up to one-third of patients with MASLD, the disease advances to metabolic dysfunction-associated steatohepatitis (MASH) which, in addition to steatosis, comprises inflammation and hepatocellular ballooning that can result in progressive fibrosis as a result of hepatic stellate cell (HSC) activation (5). Despite the recent licensing of the liver-targeted selective thyroid hormone receptor-β agonist resmetirom (6), there remains a paucity of effective pharmacological treatments for the disorder (7); this deficit is a consequence, in part, of the incomplete understanding of the underpinning pathogenic mechanisms. Progression of MASH and fibrosis is associated with an increased risk of life-threatening conditions such as hepatocellular carcinoma, cirrhosis (8), and its complications, some of which relate to increased portal vein pressure (PVP) (e.g. ascites and esophageal varices) (9). Thus, MASH, cirrhosis, and associated portal hypertension represent clear unmet medical needs, and a better understanding of pathogenesis is required to develop effective treatments.

C-type natriuretic peptide (CNP) plays an important local role in maintaining cardiac and vascular homeostasis and offsetting the development of cardiovascular disease; these cytoprotective actions are mediated via two cognate receptors, namely natriuretic peptide receptor (NPR)-B, which possesses transmembrane guanylyl cyclase activity, and G-protein-coupled NPR-C (10–13). Indeed, many of the cardio- and vasoprotective actions of endogenous CNP, including antiproliferative effects on smooth muscle cells, vasorelaxation, inhibition of leukocyte recruitment, and antifibrotic capacity (10, 11), might be anticipated to counteract the key drivers of MASH and cirrhosis, including portal hypertension, hepatic inflammation, HSC proliferation, and hepatic fibrosis; indeed, there is increasing recognition that MASH is a vascular disease of the liver (14). Pharmacological or genetic delivery of CNP supports such a concept. For example, endothelial over-expression of CNP reduces obesity-induced steatosis and hepatic inflammation (15, 16), and CNP infusion in bile duct-ligated rats reduces PVP, albeit using radiolabeled microspheres (17). Exogenous CNP also reduces inflammation and fibrosis in several organs including the lung (18), heart (11, 19, 20), and kidney (21) and exerts antiproliferative effects on human HSC (22). Furthermore, we have recently established a pivotal physiological role for CNP in maintaining metabolic homeostasis, including regulation of thermogenesis, adipogenesis, and glucose handling (23). Each of these facets of CNP biology hints that the peptide may represent an intrinsic defense mechanism to prevent the development of MASH and other liver-related diseases and that targeting CNP signaling may offer a new, effective therapeutic avenue. This notion is given further credence by the reports of altered plasma levels of CNP/NT-proCNP in human patients with cirrhosis, that correlate with disease severity (24–27) and increased CNP mRNA expression in experimental models of carbon tetrachloride (CCl_4_)-driven hepatic fibrosis (28). However, a potential biological role for CNP (and its cognate receptors, NPR-B and NPR-C) in counteracting the development of chronic liver diseases and their complications (e.g. portal hypertension), and as a potential therapy for such disorders, remains to be established.

Herein, we utilized a unique mouse model with global, inducible deletion of CNP (23) to study the effects of CNP deficiency in the progression of MASH, hepatic fibrosis, and portal hypertension using two etiologically distinct experimental models. We also explored the prospective benefit of CNP administration to reverse liver damage in this disease setting and investigated whether circulating CNP concentrations are altered in patients with liver disease.

## Materials and methods

### In vivo experimentation

All animal procedures were conducted in accordance with the UK Home Office Animals (Scientific Procedures) Act of 1986, adhered to Animal Research: Reporting of In Vivo Experiments guidelines, and were approved by the Queen Mary University of London Animal Welfare and Ethical Review Board. Animals were housed in a temperature-controlled environment with a 12-h light–dark cycle. Food and water were accessible ad libitum. For in vivo experimentation, animals were randomly assigned to interventions and the experimenter blinded to treatment wherever feasible. For cell- and tissue-based studies, interventions were randomly assigned but the experimenter was not blinded to treatment.

Male wild-type (WT or ^+/+^; C57BLK6J; 5–8 weeks of age), global CNP (23) (gbCNP^−/−^), and global NPR-C (29) (NPR-C^−/−^; a kind gift of Prof O. Smithies, University of North Carolina at Chapel Hill) knockout mice were used in this study; refer to the above citations for strain generation, confirmation of gene deletion, and baseline characterization. Male animals were utilized for this study because they have a higher prevalence of MASLD/MASH during fertile age (30). Administration of tamoxifen to CNP^flox/flox^ animals at an age of 5 weeks resulted in global deletion of CNP (gbCNP^−/−^), compared with WT (i.e. gbCNP^+/+^) littermates, as we have previously described (23).

### Disease models

#### Steatohepatitis

At 6 weeks of age, mice were switched to a choline-deficient adjusted amino acid (CDAA) diet (Research Diets, New Brunswick, USA) containing 60% kcal from fat, 0.1% methionine, and no added choline for a total of 12 weeks, established to recapitulate histological features of steatohepatitis and fibrosis with elevated serum transaminases (31). Animals initially lost weight (<10%) but over the time course of the diet increased in weight with no difference between genotypes (Δ body weight: WT = 4.54 ± 1.35 g and gbCNP^−/−^ = 4.16 ± 0.50 g; *P* = 0.7714). Mice were sacrificed at 18 weeks of age, and the livers were dissected and formalin-fixed or snap-frozen for further analyses. In some cases, to test the prophylactic effects of pharmacological CNP administration, a mini-pump (1004, Alzet, Cupertino, CA, USA) was implanted subcutaneously to deliver 0.2 mg/kg/day of the peptide from 4 weeks after the start of the CDAA diet in WT mice. Alternatively, to test the potential of CNP to reverse established disease and to simulate a change in lifestyle post-diagnosis of MASH, after 12 weeks on a CDAA diet mice were switched to standard chow (LabDiet, St. Louis, USA) for 2 weeks, during which time the animals were randomly assigned to a control group or a treatment group that received CNP (0.2 mg/kg/day; s.c.; as above). A graphical summary of the mouse models is depicted in Fig. S1.

#### Steatosis and hepatic fibrosis

WT and gbCNP^−/−^ mice were administered CCl_4_ at 9 weeks of age (i.e. 3 weeks after *Nppc* gene deletion with tamoxifen). CCl_4_ was diluted in corn oil (1:5, v/v) and injected at a dose of 0.5 µL/g of body weight three times a week for 4 weeks (i.e. 12 injections) to induce fibrosis (Fig. S1) (31). Mice were sacrificed 48–72 h after the final injection.

### Phenotypic characterization of liver disease models

Provision of a CDAA diet caused an increase in liver and spleen size compared with animals receiving standard chow and induced an increase in plasma alanine aminotransferase (ALT) and aspartate aminotransferase (AST) concentrations, indicative of liver damage (Fig. S2A–E). These changes were accompanied by increased steatosis, immune cell infiltration into the liver, and hepatic fibrosis (Fig. S2G–J). Discontinuing the CDAA diet and switching the mice to standard chow for 2 weeks, mimicking lifestyle changes in response to a MASH diagnosis, reversed several indices of disease severity including liver damage (i.e. plasma ALT and AST concentrations), steatosis, and inflammatory cell infiltrate (Fig. S2D, E, H, and I). Mirroring these observations, treatment with CCl_4_ caused an increase in plasma ALT concentration, steatosis, immune cell infiltration, and hepatic fibrosis albeit in the absence of liver or spleen enlargement (Fig. S2B, C, D, and H–J). In each disease model, the MASLD activity score was significantly higher than animals fed standard chow (Fig. S2K). These data confirm that the experimental conditions induced injury comparable to those found in MASH and fibrosis. Moreover, switching CDAA to a standard chow diet showed signs of a reduction in disease severity, as is apparent in the patient population with MASLD in response to improved dietary regimens (32).

### Assessment of steatohepatitis and hepatic fibrosis

Standard biochemical assays were used to determine plasma ALT, AST, and CNP concentrations, and routine histological analyses were employed to assess fibrosis (picrosirius red staining), immune cell infiltrate (F4/80 immunostaining), and ballooning (hematoxylin and eosin [H&E]). The development of steatosis was followed by ultrasound and endpoint echo MRI. Histological MASLD activity score (33) was used to evaluate the degree of severity of steatohepatitis.

#### Imaging

Liver tissue and portal vein were imaged using ultrasound technology at baseline and weekly (CCl_4_-treated mice) or fortnightly (CDAA-treated mice) using a 3100 high-frequency system (VisualSonics Inc., Toronto, ON, Canada) equipped with a MS550D (40-MHz) probe (Fig. S1). Images were taken at the level of the kidney in transversal orientation, with the focus point at the depth of the portal vein. Mice were kept under anesthesia (1.5% isoflurane in O_2_) on a heated mat for the duration of the imaging session. Images were taken under the same settings (frequency = 40 MHz, frame rate = 65 fps, gain = 30 dB, dynamic range = 65 dB, width = 12.08 mm, and depth = 14.00 mm) for every mouse. Portal vein area and liver steatosis were measured using Vevo LAB software 3.0.0 from B-mode images (VisualSonics Inc.). To evaluate steatosis development, pixel intensity from liver tissue was normalized to the renal cortex. This served as an internal reference as kidney pixel intensity remains constant throughout the evolution of liver steatosis. Areas with shadows from other organs or bone were discarded from the analyses.

Endpoint steatosis was measured using an EchoMRI-130 system (EchoMRI LLC, Houston, USA) equipped with an antenna for the measurement of tissue. Three independent measurements of fat and lean mass were taken, and the average of fat mass was normalized to the total weight of the tissue.

#### Serum biochemistry

Serum levels of ALT and AST were measured using a commercially available ELISA (Abcam, Cambridge, United Kingdom) in heparinized plasma obtained by cardiac puncture. Plasma CNP concentrations were also determined by a commercially available ELISA (Phoenix Pharmaceuticals, Karlsruhe, Germany) following extraction with C18 columns (10).

#### Histology

Picrosirius red (PSR) and H&E staining were performed in 4 µm formalin-fixed, paraffin-embedded liver sections from the left lobe using standard protocols. Blinded histological scoring of the level of steatosis, lobular inflammation, and hepatocellular ballooning was performed using the MASH Clinical Research Network criteria (33) by two independent investigators. Inflammatory cell count and fibrotic area were analyzed with Image Fiji software (ImageJ, NIH, USA).

Immunohistochemistry for F4/80 (murine macrophage) was performed following epitope retrieval in citrate buffer (10 mM citric acid, pH 6.0) and the inhibition of endogenous peroxidase blocking solution (3% H_2_O_2_). Slides were then permeabilized with a solution of Tween 20 (0.1%) and blocked (10% goat serum/5% bovine serum albumin/phosphate buffered saline) for 60 min. The tissue sections were then incubated with a primary antibody (1:200 dilution) against F4/80 (Bio-Rad, California, USA) overnight at 4°C. Sections were then incubated with a biotinylated secondary antibody and the streptavidin–horseradish peroxidase and finally developed with diaminobenzidine solution for 3 min.

#### In vivo PVP

In vivo PVP was determined in anesthetized animals (a flow of 2% isoflurane and 0.3 mL/min oxygen) by catheterization. To expose the portal vein, a small laparotomy was performed and the mesenterium was placed in a wet sterile gauzed for the duration of the surgery. The extra hepatic portal vein was located and isolated from the surrounding adipose tissue. Next, side branches and the distal end of the superior mesenteric vein were ligated to prevent bleeding. A SPR-671 Millar catheter (ADInstruments, Dunedin, New Zealand) previously calibrated was introduced through the superior mesenteric vein and advanced to the portal vein. After securing the catheter, PVP was recorded for a minimum of 5 min and traces analyzed using PowerLab software (ADInstruments).

#### In vitro portal vein reactivity

Portal vein reactivity was measured on a wire myograph (Danish Myotechnology, Hinnerup, Denmark). Briefly, 2 mm portal vein sections without side branches were mounted using 40 µm tungsten wires in dual-chamber wire myographs containing Krebs–Henseleit buffer solution kept at 37°C and gassed with 95% O_2_/5% CO_2_. A basal tension of 0.4 g was applied, and the vessels were left to equilibrate for 45 min. After testing the vessel viability with a single dose of KCl (80 mM), concentration–response curves to the vasoconstrictors phenylephrine (1 nM–100 μM) and U46619 (1 nM–1 μM), and the vasodilators acetylcholine (1 nM–10 μM), CNP (1 nM–1 μM), and the selective NPR-C agonist (34) cANF^4–23^ (1 nM–1 μM) were constructed and analyzed using PowerLab software (ADInstruments).

#### In vitro HSC culture

HSCs were isolated by retrogradely perfusing the liver in situ with a solution of collagenase/pronase via the inferior vena cava, then excised, and further digested in vitro for 25 min. After washing the pellet with Gey's balanced salt solution (GBSS, Sigma, United Kingdom), HSCs were isolated by density gradient-mediated separation. Cells thereby obtained were washed several times with GBSS and maintained in Dulbecco's modified Eagle's medium (Gibco, ThermoFisher Scientific, United Kingdom) with 10% fetal bovine serum (Sigma, United Kingdom) and 1% penicillin/streptomycin (Gibco, ThermoFisher Scientific). HSC proliferation was measured on days 0, 4, 7, and 10 using a WST-8-based assay kit (Cayman, Ann Arbor, MI, USA) in the absence or presence of CNP or the selective NPR-C agonist cANF^4–23^ (both 100 nM; replenished every 24 h). At day 10, HSC cell lysates were collected to evaluate protein expression (see below).

### Western blotting

Frozen tissue was homogenized (Precellys, Montigny-le-Bretonneux, France) in a sodium dodecyl sulfate (SDS) buffer (50 mM Tris base, 10% glycerol, 2% SDS) containing protease and phosphatase inhibitors (Roche, Basel, Switzerland). Equal amounts of protein were resolved by 10% SDS–polyacrylamide gel electrophoresis and transferred to a polyvinylidene fluoride membrane, which was incubated overnight with the primary antibodies (1:1,000): phospho-Smad3 (p-Smad3) (Abcam), Smad3 (Abcam), α-smooth muscle actin (Abcam), p-extracellular signal-related kinase 1/2 (ERK1/2; Cell Signaling Technology, MA, USA), ERK1/2 (Cell Signaling Technology), or glyceraldehyde-3-phosphate dehydrogenase (1:4,000; ThermoFisher Scientific). After incubation with the appropriate secondary antibodies, membranes were developed by chemiluminescence (Millipore, United Kingdom) in a chemiluminescence imaging system (GeneGnome XRQ, Syngene, United Kingdom). Densitometry analyses were performed with Image Fiji software (ImageJ).

### mRNA quantification

Total mRNA was isolated from the snap-frozen liver tissue using RNeasy Lipid Tissue Mini Kit (Qiagen, United Kingdom) following the manufacturer's instructions. cDNA was transcribed from 1 μg of mRNA after confirmation of its quality and quantity by absorption ratios at 260/280 nm and 260/230 nm using a NanoDrop spectrophotometer (Thermo Scientific, MA, USA). qRT-PCR of target gene expression was assayed using PowerUp SYBR Green (Applied Biosystems, Life Technologies Ltd, United Kingdom), and the gene-specific primers listed in Table S1 using a Bio-Rad CFX96 Connect Real-Time PCR detection system (Bio-Rad, CA, USA). The relative expression level is expressed as fold change compared with the WT expression by the 2^−ΔΔCt^ method. *Rpl-19* was used as an internal invariant control. All qRT-PCRs were performed in duplicate, and 10 ng of cDNA was used per reaction.

### Clinical sample analysis

We included healthy controls without any liver disease diagnosis or normal liver on histology, patients with compensated MASH cirrhosis (histological or radiological evidence of cirrhosis and no clinical features of decompensation), and patients with decompensated cirrhosis (radiological evidence of cirrhosis and ascites requiring paracentesis) (Table S2). Patients with decompensated cirrhosis who had hepatocellular carcinoma, viral hepatitis, or alcohol-related cirrhosis with alcohol consumption in the last 6 months were excluded. Demographic data obtained included sex, age, and ethnicity. Clinical and laboratory data obtained include type 2 diabetes status, platelet count, serum ALT concentration, and serum AST concentration. Transient elastography was performed according to standard clinical practice (reliable liver stiffness result based on successful reading rate >60% and interquartile range of all readings <30% of the median). Transient elastography was not performed in patients undergoing paracentesis.

### Study approval

Samples were obtained from patients over the age of 18 years attending hepatology outpatient clinics for paracentesis at Barts Health NHS Trust or for elective bariatric surgery at Homerton University Hospital. All participants provided written informed consent at enrollment, and the full study protocol received ethical approval from the London-Brent (18/LO/1759), Wales (14/WA/1142), and London-Bromley (20/LO/123) Research Ethics Committees.

### Statistical analysis

All data are expressed as mean ± SEM. Statistical analyses were performed using GraphPad Prism (version 9, GraphPad Software, CA, USA) after confirming normal distribution of the data using a Shapiro–Wilk test. Two-tailed, unpaired Student's t test and one-way or two-way ANOVA followed by a Šídák multiple comparisons test (with adjustment for multiplicity) were used where appropriate. Statistical significance was defined as *P* < 0.05, and the *P* values presented in each figure indicate all comparisons undertaken.

## Results

### CNP levels are lower in patients with cirrhosis and reduced further in individuals with clinically significant portal hypertension

**Fig. 1.**
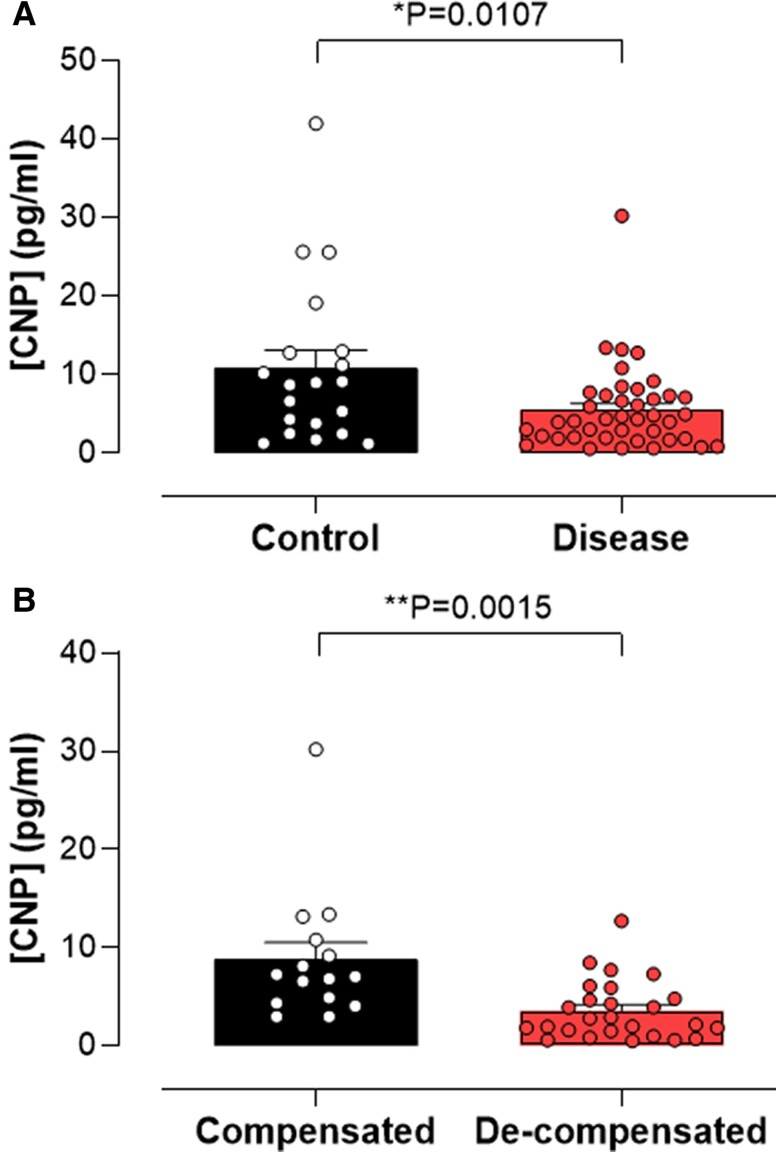
Plasma CNP concentrations are reduced in patients with MASLD. Plasma CNP concentration in control patients or those with cirrhosis (A) and comparison of plasma CNP concentration between patients with compensated and decompensated cirrhosis (B). Data are represented as mean ± SEM. Statistical analysis by two-tailed Student's t test.

Plasma CNP concentrations were significantly reduced in patients with cirrhosis in comparison with controls (Fig. 1A); moreover, there was a significant reduction in plasma CNP concentration in patients who were decompensated with clinically significant portal hypertension (Fig. 1B), compared with those with compensated cirrhosis. These data indicate that reductions in circulating CNP are associated with cirrhosis and portal hypertension in human disease.

### Enhanced hepatic fibrosis and portal hypertension in gbCNP^−/−^ mice

**Fig. 2.**
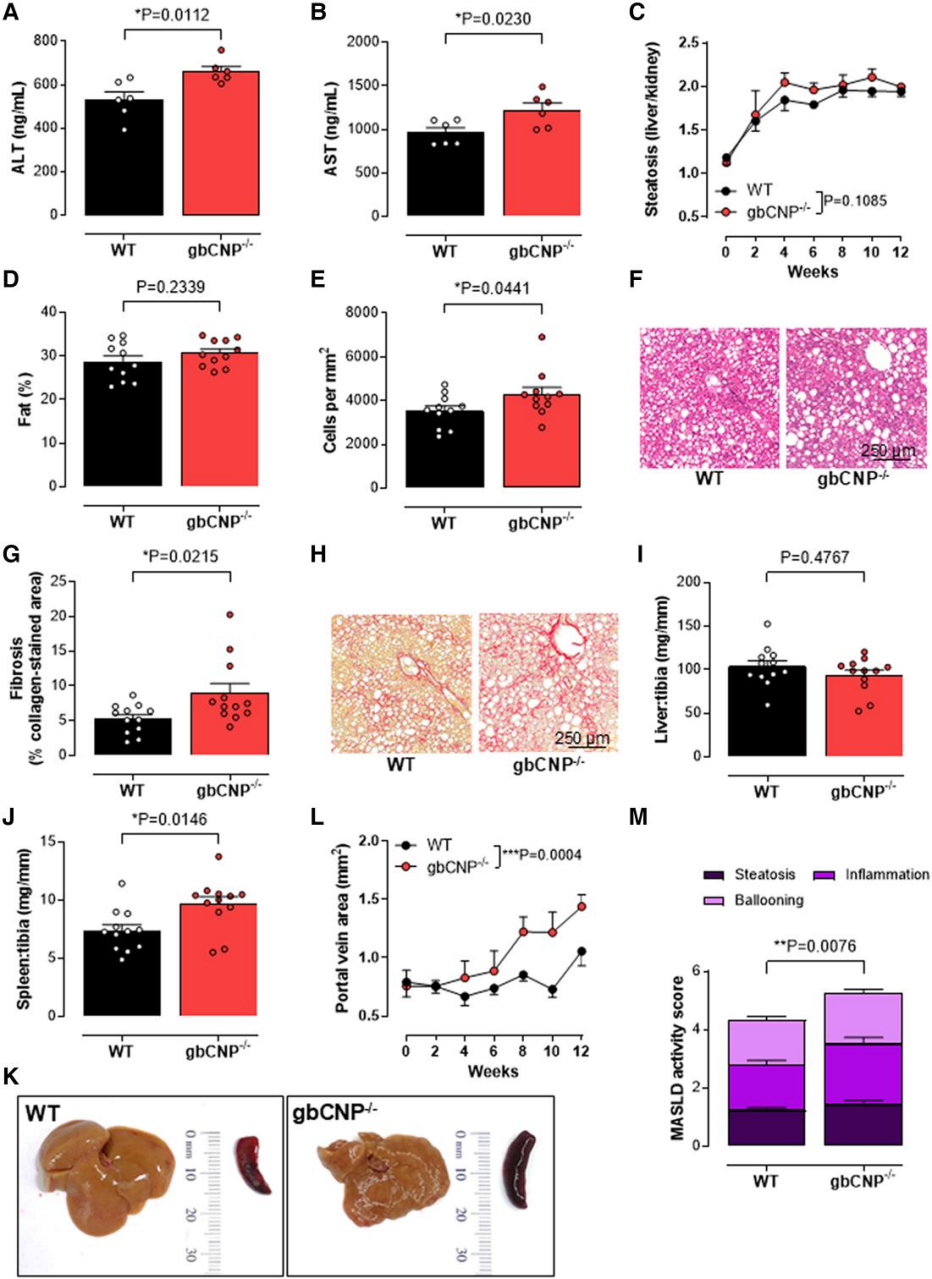
Exaggerated CDAA-induced hepatic fibrosis in gbCNP^−/−^ mice despite maintained steatosis. Plasma ALT (A) and AST (B) concentrations, steatosis (C), liver fat content (D), hepatic inflammatory cell density (E), representative H&E images showing inflammatory and immune cell ingression (F), fibrosis (% positive collagen-stained area) (G), representative images of fibrotic burden by picrosirius red staining (H), liver (I) and spleen (J) weight, representative images of the liver and spleen (K), portal vein area (L), and MASLD activity score (M) in WT and global C-type natriuretic peptide knockout (gbCNP^−/−^) mice fed a CDAA diet for 12 weeks. Data are represented as mean ± SEM. Statistical analysis by two-tailed Student's t test (A, B, D, E, G, I, J, M) or two-way ANOVA Šídák’s multiple comparisons test (C, L). Each statistical comparison undertaken has an assigned *P* value (adjusted for multiplicity).

**Fig. 3.**
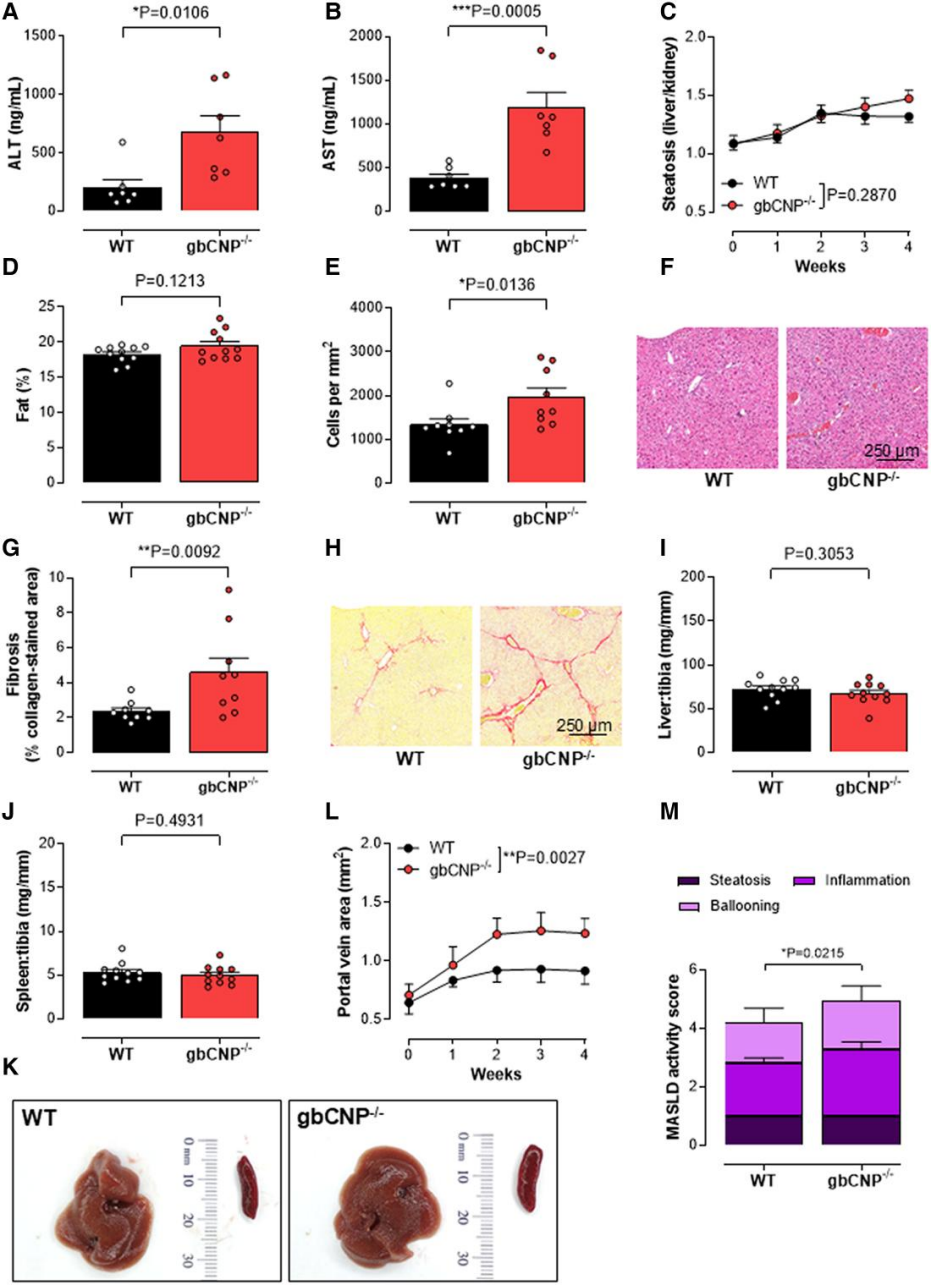
Exaggerated CCl_4_-induced hepatic fibrosis in gbCNP^−/−^ mice despite maintained steatosis. Plasma ALT (A) and AST (B) concentrations, steatosis (C), liver fat content (D), hepatic inflammatory cell density (E), representative H&E images of inflammatory and immune cell ingression (F), fibrosis (% positive collagen-stained area) (G), representative images of fibrotic burden by picrosirius red staining (H), liver (I) and spleen (J) weight, representative images of the liver and spleen (K), portal vein area (L), and MASLD activity score (M) in WT and global C-type natriuretic peptide knockout (gbCNP^−/−^) mice administered CCl_4_ for 4 weeks. Data are represented as mean ± SEM. Statistical analysis by two-tailed Student's t test (A, B, D, E, G, I, J, M) or two-way ANOVA (C, L). Each statistical comparison undertaken has an assigned *P* value (adjusted for multiplicity).

In order to model deficits in CNP signaling in patients with hepatic fibrosis and portal hypertension, we characterized the phenotype of gbCNP^−/−^ mice in experimental models of hepatic fibrosis. gbCNP^−/−^ mice fed a CDAA diet exhibited increased hepatic fibrosis, inflammatory/immune cell infiltration into the liver, evidence of hepatocellular damage (i.e. plasma AST and ALT concentrations), and increased spleen weight and portal vein area compared with WT littermates (Figs. 2A, B, E–H, and J–L and S3A); though, steatosis and liver size were unchanged between genotypes (Fig. 2C, D, and I). Overall, gbCNP^−/−^ caused a significant increase in disease severity and, in the small number of mice fed a CDAA diet, progression to cirrhosis was more commonly observed in gbCNP^−/−^ animals compared with WT (21.4 versus 7.1%, respectively). We reasoned that the greater severity in gbCNP^−/−^ mice indicates CNP acts as an endogenous defense mediator that offsets the development of liver injury. To determine whether this was common to other modes of liver injury, a second CCl_4_-induced liver injury model was utilized. As with the CDAA model, gbCNP^−/−^ mice had greater hepatic fibrosis, immune cell infiltration into the liver, evidence of hepatocellular damage (i.e. plasma AST and ALT concentrations), and portal vein area compared with WT littermates (Figs. 3A, B, E–H, and L and S3B); however, and as expected, steatosis and liver and spleen weight were not significantly different (Fig. 3C, D, and I–K).

**Fig. 4.**
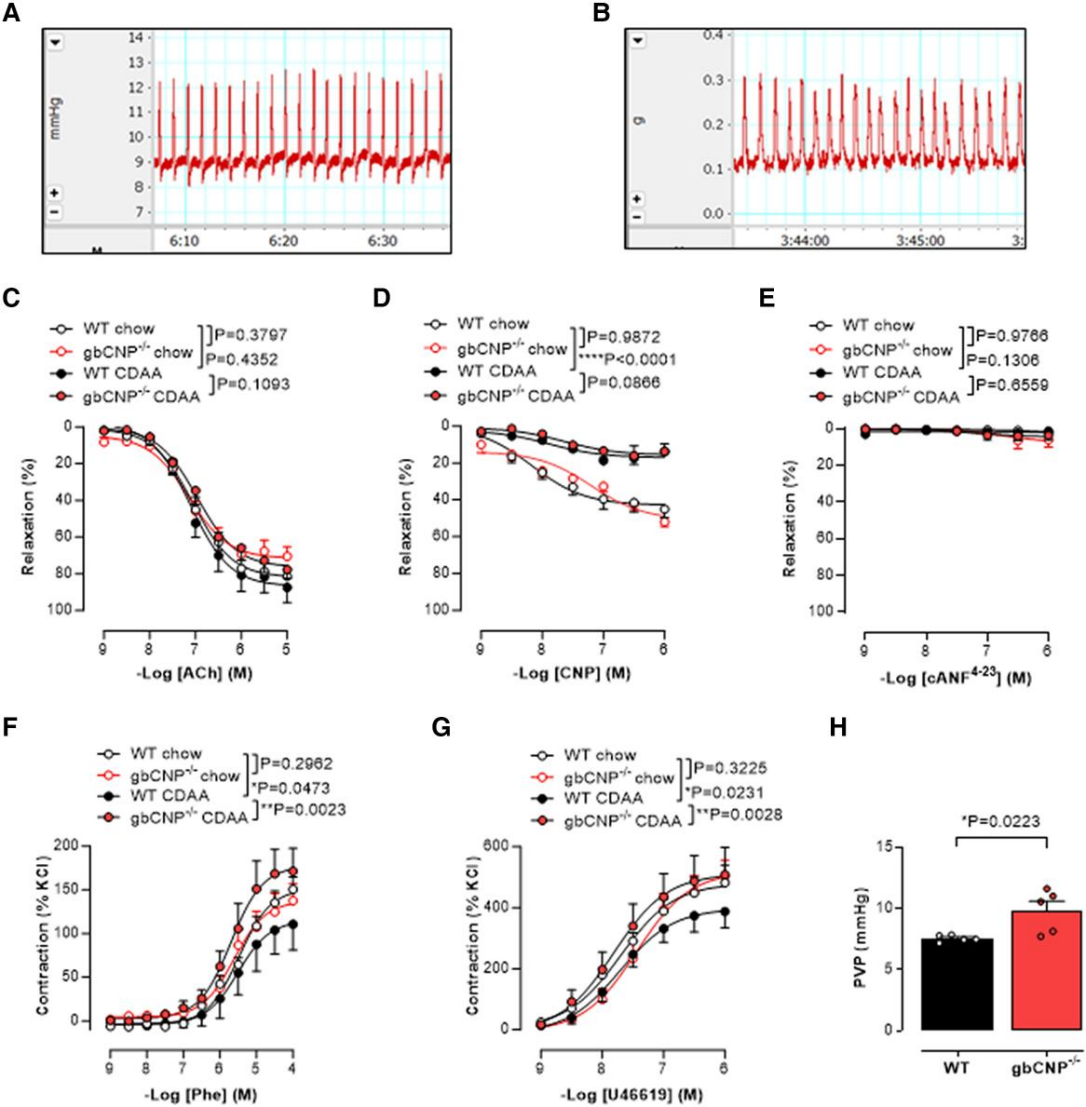
Increased PVP and reduced portal vein sensitivity to CNP in gbCNP^−/−^ mice. Representative traces depicting the spontaneous reactivity of the portal vein in situ (A) and ex vivo (B). Ex vivo vascular reactivity to acetylcholine (ACh; C), CNP (D), cANF^4–23^ (E), phenylephrine (Phe; F), and U46619 (G), in WT or global C-type natriuretic peptide knockout (gbCNP^−/−^) mice fed standard chow or CDAA for 12 weeks and PVP (H) in mice fed CDAA. Data are represented as mean ± SEM. Statistical analysis by two-way ANOVA Šídák’s multiple comparisons test (C, D, E, F, G) or two-tailed Student's t test (H). Each statistical comparison undertaken has an assigned *P* value (adjusted for multiplicity).

Portal hypertension can be a consequence of structural and/or vasoactive changes that increase intrahepatic vascular resistance. We found the portal vein diameter in gbCNP^−/−^ mice with hepatic fibrosis to be significantly higher than in mice WT (although no difference at baseline; Fig. 2L); therefore, we sought to confirm this was associated with a higher PVP and/or altered portal vein reactivity in response to pharmacological stimulation. Portal vein segments harvested from WT mice fed a CDAA diet were less sensitive to CNP (and the vasoconstrictors phenylephrine and U46619 but not the NPR-C-selective agonist cANF^4–23^; Fig. 4A–G) compared with animals fed standard chow; in contrast, relaxations to ACh were unaltered, indicative of preserved endothelial function (Fig. 4C). These changes are almost certainly a consequence of the pathology induced by the CDAA diet per se (rather than CNP gene deletion), since WT and gbCNP^−/−^ control mice fed a standard chow diet had an analogous, greater responsiveness to CNP and vasoconstrictors (Fig. 4D–G). CDAA-fed gbCNP^−/−^ animals with hepatic fibrosis had a higher in vivo PVP compared with WT controls, mirroring the altered reactivity in isolated vein segments. This occurred without any change in sensitivity to endothelium-dependent dilatation in response to ACh, or to CNP or cANF^4–23^, but there was increased response to vasoconstrictors (Fig. 4H–M). Interestingly, vasorelaxant responses to the selective NPR-C agonist (34) cANF^4–23^ were essentially absent in portal veins from WT and gbCNP^−/−^ mice (Fig. 4E and K), implying that the vasorelaxant actions of CNP in this vessel are not mediated by NPR-C. Similar findings with respect to endothelium-dependent dilatation, CNP responsiveness, and vasoconstrictor activity were observed in portal veins from CCl_4_-treated WT mice with hepatic fibrosis (Fig. S4A–E). There was no further difference in the vasorelaxant responses to CNP in CCl_4_-treated gbCNP^−/−^ animals compared with WT counterparts although contractile responses were increased (Fig. S4F–J). Taken together, the reduced potency of CNP in both models (and greater responsiveness to vasoconstrictors in the CCl_4_ model) of hepatic fibrosis is likely to result in increased tone in the portal vasculature and contribute to portal hypertension (albeit we have not evaluated splanchnic vasodilation herein which could also contribute).

### NPR-B versus NPR-C signaling in MASH and the therapeutic potential of targeting these pathways

**Fig. 5.**
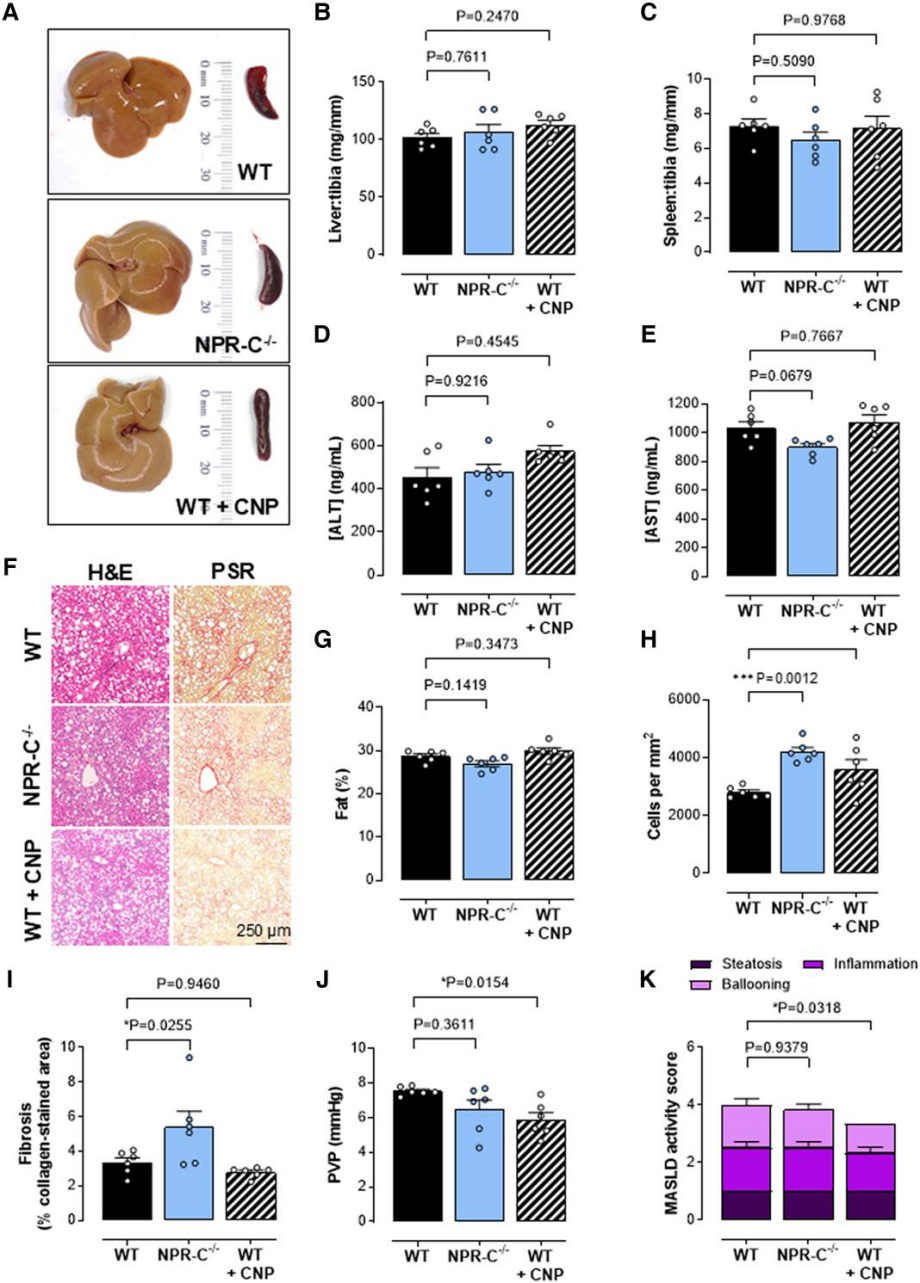
Increased liver inflammation in NPR-C^−/−^ mice and therapeutic effect of exogenous CNP. Representative images of the liver and spleen (A), liver (B), and spleen (C) weight normalized to tibia length and plasma ALT (D) and AST (E) concentrations, representative images of inflammatory and immune cell ingression (H&E staining) and fibrosis (picrosirius red staining; F), liver fat content (G), hepatic inflammatory cell density (H), fibrosis (% positive collagen-stained area) (I), PVP (J), and MASLD activity score (K) in 12-week CDAA-fed WT, global NPR-C knockout (NPR-C^−/−^) mice, and WT mice receiving CNP (0.2 mg/kg/day, s.c.; from weeks 5 to 12). Data are represented as mean ± SEM. Statistical analysis by one-way ANOVA Šídák’s multiple comparisons test. Each statistical comparison undertaken has an assigned *P* value (adjusted for multiplicity).

**Fig. 6.**
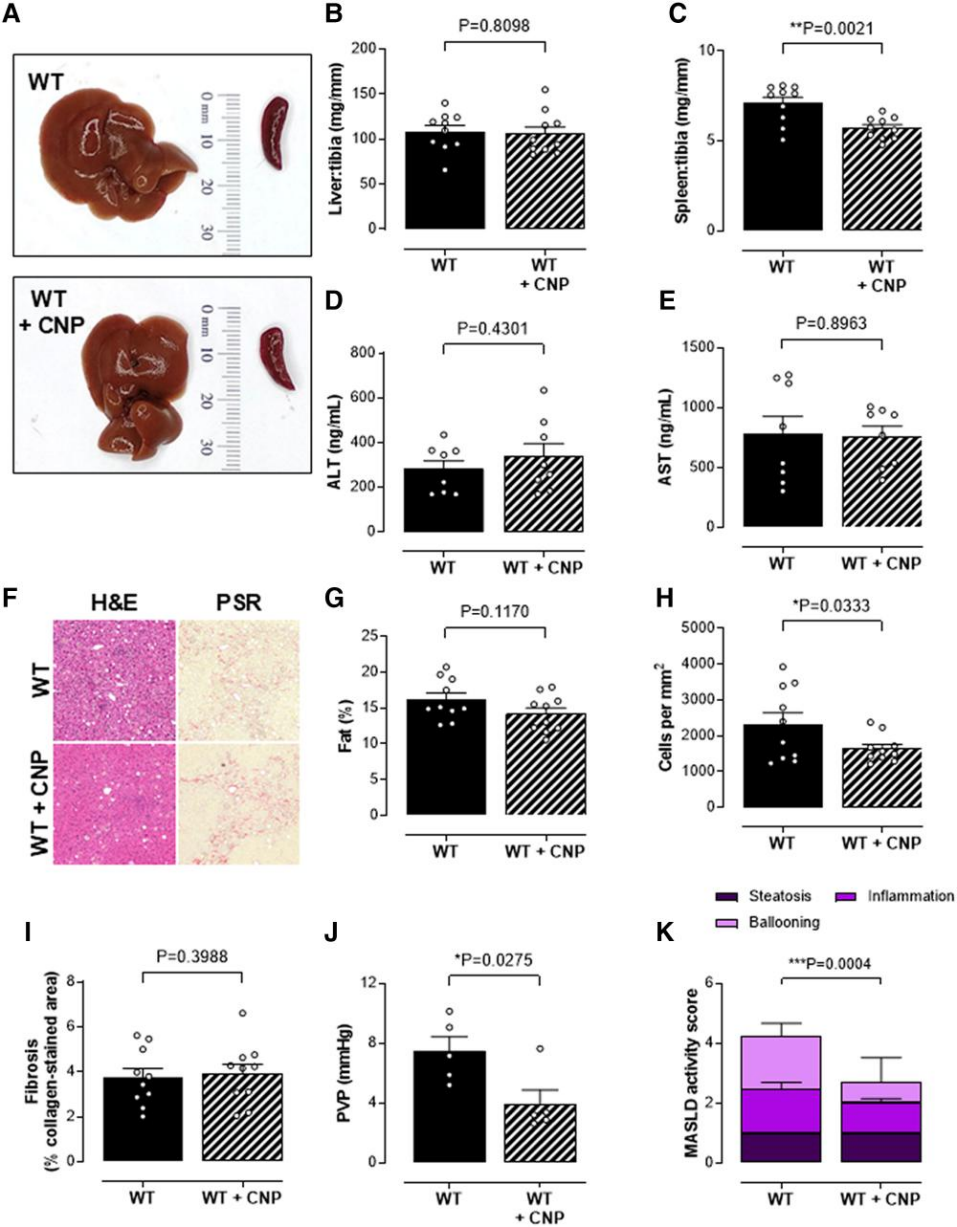
CNP treatment reverses the severity of NASH in combination with a switch to normal chow. Representative images of the liver and spleen (A), liver (B), and spleen (C) weight normalized to tibia length and plasma ALT (D) and AST (E) concentrations, representative images of immune cell ingression (H&E staining) and fibrosis (picrosirius red staining; F), liver fat content (G), hepatic inflammatory cell density (H), fibrosis (% positive collagen-stained area) (I), PVP (J), and MASLD activity score (K) in control WT mice and WT mice receiving CNP (0.2 mg/kg/day, s.c.; from weeks 12 to 14; WT + CNP) fed a CDAA diet for 12 weeks and then switched to normal chow for 2 weeks. Data are represented as mean ± SEM. Statistical analysis by two-tailed Student's t test. Each statistical comparison undertaken has an assigned *P* value (adjusted for multiplicity).

To determine which cognate receptor(s) (i.e. NPR-B versus NPR-C) underpin the beneficial actions of endogenous CNP in MASH and fibrosis, we explored disease severity in NPR-C^−/−^ mice. Genetic deletion of NPR-C resulted in worsening of fibrotic burden and immune cell infiltrate in mice fed a CDAA diet (Fig. 5A–I). However, the fact that NPR-C^−/−^ mice had a similar PVP compared with WT controls confirmed the likely importance of NPR-B in the pathogenesis of portal hypertension in this model (Fig. 5J). To determine whether pharmacologically targeting such pathways might represent a novel therapeutic strategy, we administered exogenous CNP to WT mice. Administration of CNP as a prophylactic treatment was effective in lowering PVP (Fig. 5J), and therapeutic infusion of CNP for 2 weeks after switching from a CDAA diet to standard chow additionally reduced intrahepatic inflammatory and immune cell density and spleen size, but not fibrosis (Fig. 6A–K).

### Markers of inflammation, fibrosis, and tissue remodeling are up-regulated in mice lacking CNP, but reversed by CNP treatment

To evaluate which pathogenic pathways are beneficially regulated by endogenous and exogenous CNP in MASH and fibrosis, the mRNA expression of key markers of inflammation, fibrosis, tissue remodeling, and CNP signaling was evaluated.

In WT mice, both CDAA diet and CCl_4_ administration induced up-regulation of NPR-C, which was partially reversed by switching to standard chow diet for 2 weeks and down-regulation of NPR-B expression that was not reversed after switching to chow diet for 2 weeks in WT animals. The expression of CNP mRNA in the liver was lower in CDAA-fed mice but unchanged following CCl_4_ administration (Fig. S5A and Table S3). Despite this, plasma CNP concentrations were increased in both models (Fig. S2F), which may suggest extrahepatic sites of CNP release following liver injury. Both the CDAA diet and CCl_4_ models resulted in a significant up-regulation of several markers of inflammation, fibrosis, and tissue remodeling; most of these proinflammatory (i.e. IL-1β, IL-6, TNFα), profibrotic (i.e. TGFβ, Col1a1, and Col1a2), and proremodeling (i.e. TIMP-1 and MMP-13) pathways were up-regulated after switching from CDAA to a standard chow diet (Fig. S5A–C and Table S3). In mice fed standard chow and those receiving CCl_4_, macrophage infiltration was not significantly increased and very diffuse in nature (Fig. S5D). In contrast, CDAA-fed mice showed significant macrophage accumulation surrounding enlarge lipid-laden hepatocytes (Fig. S5D).

**Fig. 7.**
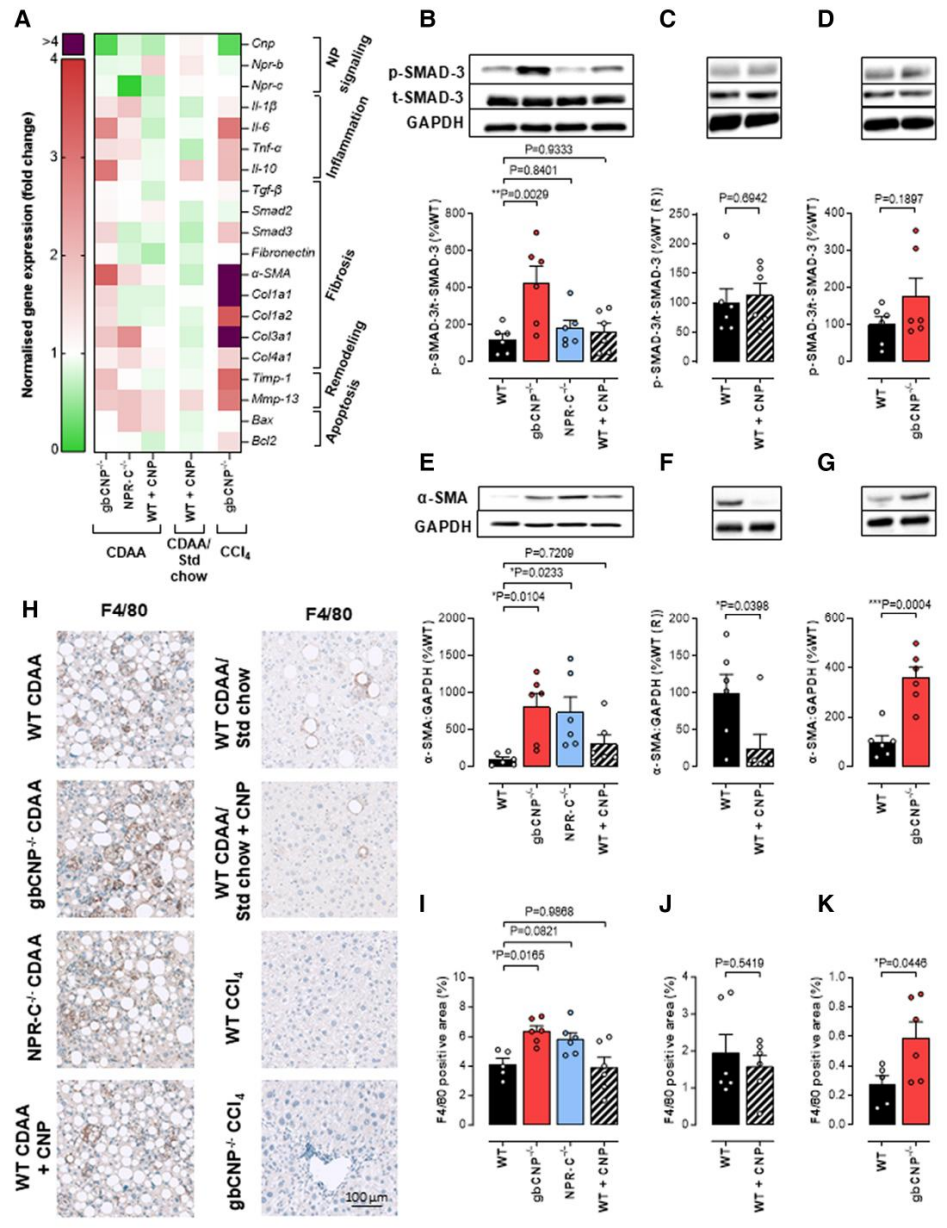
Markers of inflammation, fibrosis, and tissue remodeling are up-regulated in mice lacking CNP, but reversed following CNP treatment. Heatmap of relative liver mRNA expression of natriuretic peptide (NP) signaling, and markers of inflammation, fibrosis, tissue remodeling, and apoptosis (A). p-SMAD-3 phosphorylation (B, C, D), αSMA protein expression (E, F, G), and macrophage infiltration (F4/80 staining, I, J, K; with representative images, H) in 12-week CDAA-fed WT, global NPR-C knockout (NPR-C^−/−^) mice, and WT mice receiving CNP (0.2 mg/kg/day, s.c.; from weeks 5 to 12). Data are represented as mean ± SEM. Statistical analysis by one-way ANOVA with Šídák’s multiple comparisons test (B, E, I) or two-tailed Student's t test (C, D, F, G, J, K). Each statistical comparison undertaken has an assigned *P* value (adjusted for multiplicity).

gbCNP^−/−^ mice had an exacerbated expression of most proinflammatory, profibrotic, and proremodeling markers studied when fed a CDAA diet or following CCl_4_ administration (Fig. 7A and Tables S4 and S5). NPR-C^−/−^ mice, in contrast, only showed up-regulated expression of a subset of these markers (Fig. 7A and Table S6), further supporting the indication from the ex vivo work above that, in concert with NPR-C-triggered signaling pathways, NPR-B (the alternate cognate receptor for CNP) is also likely to be involved in the molecular underpinnings of pathogenesis.

CNP administration as a prophylactic or reversal treatment caused an up-regulation of NPR-B but largely left CNP and NPR-C expression unaltered (Fig. 7A and Table S6). Expression of proinflammatory cytokines (e.g. IL-1β and TNFα) showed a similar pattern whether CNP was administered prophylactically or after switching to a standard chow diet, although this beneficial pharmacological action was more pronounced in the latter model (Figs. 7A and S1 and Table S6). Moreover, both treatments reduced fibronectin expression, a profibrotic marker that associates with a worse outcome in obese patients with MASH (35, 36). In accord, alpha smooth muscle actin (αSMA) expression and p-Smad3 expression were both up-regulated in mice receiving a CDAA diet or CCl_4_ (Fig. 7B–G). αSMA expression was up-regulated in both gbCNP^−/−^ and NPR-C^−/−^ mice fed a CDAA diet or administered CCl_4_ and was down-regulated after CNP treatment (Fig. 7E–G), suggesting that a CNP/NPR-C pathway regulates myofibroblast activation (i.e. αSMA) in fibrosis. p-Smad3 increased in CDAA-fed mice, but not in CCl_4_-treated gbCNP^−/−^ mice fed a CDAA diet, suggesting ostensibly Smad3-independent underpinning mechanisms (Fig. 7B–D). Immunohistochemical staining for F4/80 (activated macrophages) provided further evidence corroborating the effects of CNP deletion (increased) or pharmacological administration (reduced) on inflammation (Fig. 7H–K). Importantly, mRNA expression of the proapoptotic and antiapoptotic genes Bax and Bcl2 was not influenced by genotype or CNP administration, suggesting that dysregulated apoptosis does not contribute to the genotypic differences in disease severity (Figs. 7A and S5 and Tables S3–S6).

In sum, these data suggest that loss of CNP is associated with increased hepatic inflammation which is reversed partially after switching to chow diet; this reversal process is accelerated by administration of CNP. Moreover, in contrast to the changes in the portal vasculature which appear to be exclusively NPR-B-dependent, the effects of CNP on liver injury are a combination of NPR-B- and NPR-C-triggered pathways.

### CNP exerts antimitogenic effects in HSC via NPR-B

**Fig. 8.**
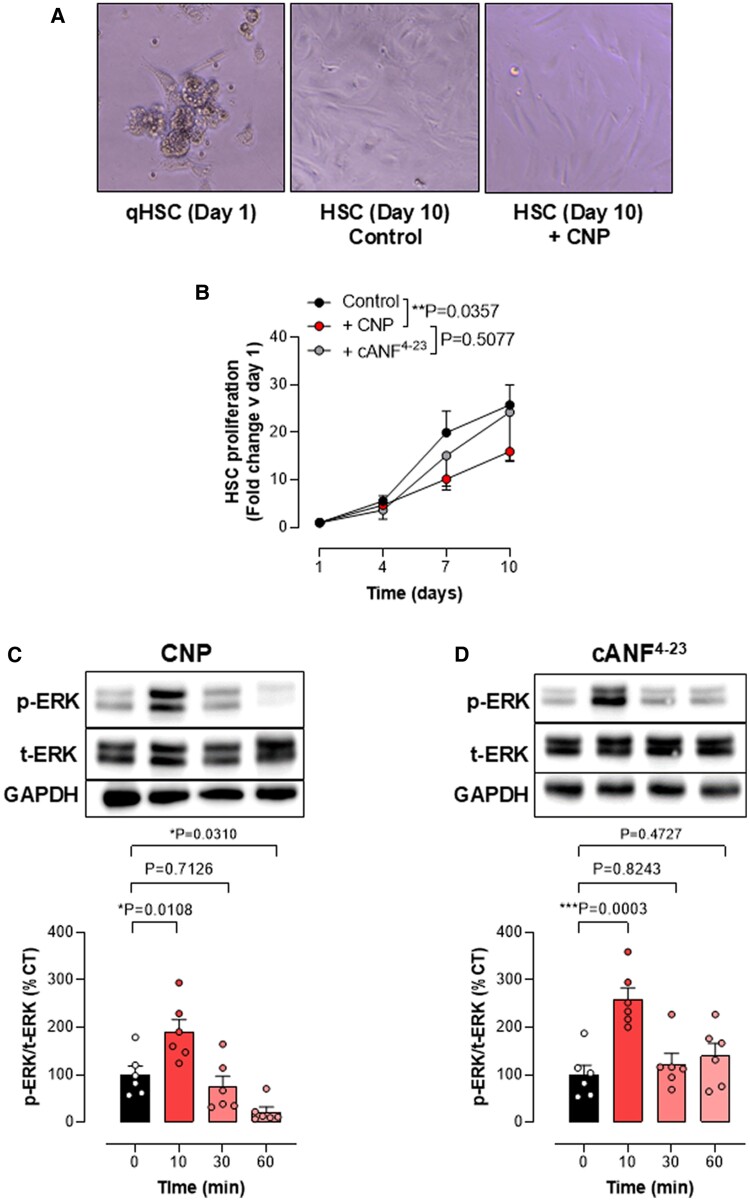
CNP exerts antimitogenic effects in HSC via NPR-B. Representative pictures of qHSC (day 1) and differentiated HSC (day 10) (A). Proliferation curve of HSC untreated (CT) or treated daily with CNP (100 nM) or cANF^4–23^ (100 nM) (B). ERK1/2 phosphorylation in HSC on the absence (CT) or after 10, 30, or 60 min after the addition of (100 nM; C) or cANF^4–23^ (100 nM; D). Data are represented as mean ± SEM. Statistical analysis by two-way repeated measures ANOVA (B) or one-way ANOVA with Šídák’s multiple comparisons test (C, D). Each statistical comparison undertaken has an assigned *P* value (adjusted for multiplicity).

In order to determine whether the specific effect of CNP in the major cell type that drives fibrosis is mediated by NPR-B or NPR-C, we studied isolated HSCs from control WT mice. Initial studies revealed that HSC express all three NPRs (Fig. S6). Upon activation, HSCs differentiate from vitamin A-storing pericytes into myofibroblasts; this is a key phenotypic change that drives hepatic damage and fibrosis. HSCs were isolated from the livers of WT animals and cultured in the presence of CNP or cANF^4–23^. By day 10, all cells had completed differentiation, as confirmed by the change in morphology, from quiescent lipid-storing cells (qHSC, day 1) to fibroblast-like morphology (HSC, day 10; Fig. 8A). In contrast to cANF^4–23^, CNP reduced HSC proliferation, suggesting that the antimitogenic effects of CNP are NPR-B mediated (Fig. 8A and B). Next, we evaluated the expression and activation of ERK1/2, a key mediator in the regulation of cell proliferation, differentiation, and cell death in HSC. Both CNP and cANF^4–23^ induced early phosphorylation after 10 min (Fig. 8C and D), but whereas CNP showed a significant reduction in ERK 1/2 phosphorylation after 60 min compared with baseline (CT), cells treated with cANF^4–23^ did not show these diminished levels of phosphorylation at this later timepoint (Fig. 8C and D). These data indicate that the sustained inhibitory effect of CNP on HSC proliferation is mediated primarily via NPR-B (since selective NPR-C agonism did not recapitulate the reduced ERK 1/2 activation). These data suggest that one of the antifibrotic pathways activated by CNP is NPR-B-dependent inhibition of HSC proliferation. Consequently, the exacerbated fibrosis and immune cell infiltrate observed in NPR-C^−/−^ mice are likely to be mediated via alternate cell types, particularly effects of CNP on inflammatory cell extravasation (10) and fibroblasts (37).

## Conclusion

The key contribution of CNP, emanating from multiple cell types including cardiomyocytes, fibroblasts, endothelial cells, and pericytes, to cardiac and vascular homeostasis is now clear; a role spanning actions on heart structure and function, vascular function, local blood flow, immune cell recruitment, platelet reactivity, and angiogenesis (10–13, 38). Herein, we employed a global, inducible CNP null mutant mouse to study the role of CNP in the development of steatohepatitis, hepatic fibrosis, and portal hypertension using well-established CDAA and CCl_4_ experimental models. gbCNP^−/−^ mice were more susceptible to developing advanced hepatocellular damage, inflammation, fibrosis amounting to cirrhosis, and perhaps mostly significantly portal hypertension. These events were dependent on both cognate receptors for CNP (i.e. NPR-B and NPR-C) and associated with a higher expression of proinflammatory and profibrotic markers; moreover, disease progression was partially reversed by the administration of CNP. Such observations establish a new and important role for endogenous CNP as a regulator of hepatic health, and they complement and extend our recent report describing CNP as a critical regulator of metabolic homeostasis (23). Moreover, these data suggest that CNP-targeted therapy might be a novel means to tackle MASH and potentially other liver disorders that lead to fibrosis and portal hypertension.

Previous work has offered contrasting findings with respect to plasma CNP concentrations in people with cirrhosis, with both higher and lower levels reported (25, 27). However, our data reveal a clear reduction in plasma CNP concentrations in patients with MASH cirrhosis compared with controls without overt liver disease and, in addition, that patients with decompensated cirrhosis with evidence of portal hypertension have significantly reduced circulating levels of CNP compared with patients with compensated cirrhosis. This finding supports the concept that endogenous CNP offsets the pathogenesis of MASLD/cirrhosis in the patient population and links deficits in CNP bioactivity to portal hypertension (i.e. lower levels in decompensated patients); moreover, these data dovetail well with correlations between CNP and markers of liver damage (i.e. ALT, AST). However, in the murine disease models studied herein, the liver expression of CNP tended to be reduced in disease, whereas plasma levels of the peptide were increased. Therefore, it may be that additional cellular sources of CNP are up-regulated to compensate for the loss of hepatic CNP production. One possibility is that the increased pressure in the portal circulation triggers the release of CNP from the endothelium, since shear stress is a key stimulus for this process (39).

Immune cell infiltration, steatosis, cell injury, and fibrosis are the key processes driving the pathogenesis of MASH. Deletion of CNP in the CDAA diet and CCl_4_ models of steatohepatitis and hepatic fibrosis results in a higher cell infiltration density, collagen deposition, and hepatocyte injury (e.g. elevated plasma transaminase concentrations) as well as an up-regulated expression of proinflammatory cytokines, profibrotic markers, and pathways driving tissue remodeling. This finding is consistent with previous reports documenting increased fibrosis and inflammation in a range of pathological models in which CNP signaling is depleted (11). Likewise, potentiating CNP pathways seems to reduce inflammation and prevent the development of fibrosis (18, 19, 21). The mechanism(s) underlying the protective actions of CNP seem to be related to the suppression of fibroblast differentiation into myofibroblasts (18), a dampening of endothelial activation and leukocyte recruitment (10), and a reduction in proinflammatory cytokine release (15, 40, 41). In accord, our work in experimental steatohepatitis and hepatic fibrosis reveals an up-regulation of the TGFβ signaling pathway and proinflammatory cytokine expression (i.e. IL-1β, IL-6, and TNFα) in mice lacking CNP; moreover, many of these pathogenic processes can be reversed by CNP treatment. Interestingly, antiinflammatory effects of CNP have been reported to be regulated via NPR-C-dependent pathways and suppression of P-selectin expression (10); this would fit well with the overt effects of CNP and NPR-C gene deletion on hepatic immune cell infiltration in vivo described herein. Such observations dovetail well with previous reports demonstrating that NPR-C signaling governs fibrosis in the heart via TGFβ and TIMP1 (42). Indeed, in this study NPR-C^−/−^ animals mirrored some of the pathological features observed in gbCNP^−/−^ mice (i.e. increased cell infiltration, fibrosis, and inflammatory markers, up-regulated αSMA protein expression, and Col3a1 mRNA), supporting the idea that NPR-C plays a role in dampening inflammation and fibrogenesis. However, NPR-C deletion did not recapitulate all the pathological changes observed in gbCNP^−/−^ mice, highlighting that CNP acting through its alternate cognate receptor, NPR-B, also slows the development of MASH and fibrosis. Indeed, we show that HSCs express both NPR-B and NPR-C and previous multiomic studies in both murine models of MASLD and human hepatic tissue suggest that multiple cell types in the liver express NPRs, including endothelial cells, fibroblasts, and hepatocytes (43, 44), implying that a multimodal effect of CNP, through both cognate NPRs on several cell types, is likely to underpin the salutary actions of the peptide in MASLD.

In contrast to fibrosis and inflammation, liver steatosis in response to the choline-deficient diet was not modified in mice lacking CNP or NPR-C, nor after CNP treatment. It has been previously reported that transgenic mice overexpressing CNP in endothelial cells (15) and mice lacking NPR-C (45) are protected from HFD-induced steatosis. However, since we and others have established that gbCNP^−/−^ and NPR-C^−/−^ mice have altered lipid metabolism, with a prothermogenic phenotype (23, 46) and the fact that the CDAA model in particular results in altered liver transport and metabolism of fatty acids, it is not surprising that gbCNP^−/−^ and NPR-C^−/−^ mice in the present study did not show any changes in liver fat deposition.

Portal hypertension and spleen size were increased in choline-deficient diet-fed gbCNP^−/−^ mice compared with WT and were reversed by pharmacological delivery of CNP. These complementary findings imply endogenous CNP regulates PVP, and this function can be recapitulated by CNP administration. However, the reduced responsiveness to CNP in the portal vein in the CDAA model is independent of genotype (i.e. there are no baseline differences in CNP sensitivity), indicating that it is the pathogenesis of steatohepatitis and fibrosis that underpin this dysfunctional signaling. The beneficial actions of CNP on portal hypertension likely comprise at least two components. The first is through changes in the architecture of the liver parenchyma. HSCs contribute to portal hypertension through increased collagen deposition and dysfunctional angiogenesis that modify the parenchymal architecture and increase vascular resistance (in tandem with greater release of vasoconstrictors) (47). Herein, we show that CNP reduces HSC proliferation to offset these structural changes, in part via NPR-B-driven inhibition of ERK1/2 phosphorylation. This fits with previous findings in human HSC (22) and might be related to the comparatively higher expression of NPR-B in human liver (48). Thus, given the exaggerated HSC proliferation and collagen deposition observed in mice lacking CNP, it is reasonable to conclude that structural changes result in increased portal pressure in vivo when CNP signaling is suppressed. Second, reduced portal vein responsiveness to CNP per se contributes to higher PVP, with NPR-B-dependent signaling also underpinning the vasorelaxant actions of CNP (as has been shown for other large vessels (49)). This multimodal ability of CNP to offset the development of portal hypertension is arguably one of the key findings of the current study and implies that pharmacological augmentation of this peptide holds promise for targeted lowering of portal pressure in patients with complications of MASLD. Current therapies used in portal hypertension include nonselective β-blockers to counteract splanchnic β_2_-mediated vasodilatation and drugs that increase nitric oxide bioavailability to promote sinusoidal vasodilatation (50). Neither of these interventions are disease modifying, whereas the multifaceted effects of CNP described herein suggest that pharmacological delivery of the peptide can reverse portal hypertension while simultaneously targeting the underlying pathogenesis. Interestingly, prophylactic administration of CNP during maintained pathogenic stimulus (i.e. CDAA diet) had little or no effect on outcome; in contrast, the application of CNP in concert with a switch to a normal diet accelerated reversal of many aspects of pathogenesis, including reversal of the portal hypertension, albeit we did not run the experiment out to endpoints such as ascites.

In conclusion, this study shows a critical role of endogenous CNP in the prevention of steatohepatitis, hepatic fibrosis, and portal hypertension, advances the understanding of the pathogenesis of the disease, and advocates targeting CNP signaling as a prospective new therapeutic approach.

## Supplementary Material

pgae579_Supplementary_Data

## Data Availability

All study data are included in the main text and Supplemental Information.
