# Supplementary material for: Endogenous C-type natriuretic peptide offsets the pathogenesis of steatohepatitis, hepatic fibrosis, and portal hypertension
Source: PNAS Nexus. 2024 Dec 30;4(1):pgae579. doi: 10.1093/pnasnexus/pgae579 (PMC11734523; doi:10.1093/pnasnexus/pgae579)
Supplement: pgae579_Supplementary_Data [file pgae579_supplementary_data.docx]

**SUPPLEMENTAL INFORMATION**

**Supplemental Table 1: Mouse primers**

| **Target gene** | **Primer sequence (5’-3’)** | **Accession codes** |
| --- | --- | --- |
| Mouse Bcl2 (*Bcl2*)  Forward | AGGCTGGGATGCCTTTGTGG | NM_009741.5 |
| Mouse Bcl2 (*Bcl2*)  Reverse | TGTTTGGGGCAGGTTTGTCG |  |
| Mouse Bax (*Bax*)  Forward | TGCAGAGGATGATTGCTGACG | NM_007527.4  NM_001411994.1  NM_001411995.1  NM_001411996.1 |
| Mouse Bax (*Bax*)  Reverse | AGCCACCCTGGTCTTGGAT |  |
| Mouse Cnp (*Nppc*) Forward | CCAACGCGCGCAAATACAAA | NM_010933.5 |
| Mouse Cnp (*Nppc*) Reverse | GCACAGAGCAGTTCCCAATC |  |
| Mouse Col1a1 (Col1a1) Forward | TCTGACTGGAAGAGCGGAGAG | NM_007742.4 |
| Mouse Col1a1 (Col1a1) Reverse | AGACGGCTGAGTAGGGAACA |  |
| Mouse Col1a2 (Col1a2) Forward | TGGATACGCGGACTCTGTTG | NM_007743.3 |
| Mouse Col1a2 (Col1a2) Reverse | CCCTTTCGTACTGATCCCGATT |  |
| Mouse Col3a1 (Col3a1) Forward | GGGAGGAATGGGTGGCTATC | NM_009930.2 |
| Mouse Col3a1 (Col3a1) Reverse | CTGGGCCTTTGATACCTGGA |  |
| Mouse Col4a1 (Col4a1) Forward | CTGGAGAAAAGGGCCAGAT | NM_009931.2 |
| Mouse Col4a1 (Col4a1) Reverse | TCCTTAACTTGTGCCTGTCC |  |
| Mouse Fibronectin (Fn1) Forward | CCGGTGGCTGTCAGTCAGA | NM_010233.2  NM_001276408.1  NM_001276409.1  NM_001276411.1  NM_001276412.1  NM_001276413.1 |
| Mouse Fibronectin (Fn1) Reverse | CCGTTCCCACTGCTGATTTATC |  |
| Mouse Il-1β (Il1b) Forward | TGCCACCTTTTGACAGTGATG | NM_008361.4 |
| Mouse Il-1β (Il1b) Reverse | TGATGTGCTGCTGCGAGATT |  |
| Mouse Il-6 (Il6) Forward | TCGTGGAAATGAGAAAAGAGTTGTG | NM_031168.2  NM_001314054.1 |
| Mouse Il-6 (Il6) Reverse | ACTCCAGAAGACCAGAGGAAA |  |
| Mouse Il-10 (Il10) Forward | GGGTTGCCAAGCCTTATCGG | NM_010548.2 |
| Mouse Il-10 (Il10) Reverse | TAGACACCTTGGTCTTGGAGCTTAT |  |
| Mouse Mmp13 (Mmp13) Forward | GACAAGCAGTTCCAAAGGCTAC | NM_008607.2 |
| Mouse Mmp13 (Mmp13) Reverse | TAGGGCTGGGTCACACTTCT |  |
| Mouse Npr-B (*Npr2*) Forward | AACGGGCGCATTGTGTATATCT | NM_173788.4  NM_001355466.1 |
| Mouse Npr-B (*Npr2*) Reverse | TCAGGATTTGGGGGTTCTCG |  |
| Mouse Npr-C (*Npr3*) Forward | CTTGGATGTAGCGCACTATGTC | NM_001039181.1  NM_008728.2 |
| Mouse Npr-C (*Npr3*) Reverse | CACAAGGACACGGAATACTC |  |
| Mouse Rpl19 (*Rpl19*) Forward | TTGGCGATTTCATTGGTCTCA | NM_009078.2  NM_001159483.1 |
| Mouse Rpl19 (*Rpl19*) Reverse | GCTTGCCTCTAGTGTCCTCC |  |
| Mouse α-Sma (Acta2) Forward | ACTACTGCCGAGCGTGA | NM_007392.3 |
| Mouse α-Sma (Acta2) Reverse | ATAGGTGGTTTCGTGGATGC |  |
| Mouse Smad2 (Smad2) Forward | AGTTGAGACCCCAGTCTTGC | NM_001311070.1  NM_001252481.1  NM_010754.5 |
| Mouse Smad2 (Smad2) Reverse | GAGACAGTTCAGCCGGAGAG |  |
| Mouse Smad3 (Smad3) Forward | CAGCATGGACGCAGGTTCT | NM_016769.4 |
| Mouse Smad3 (Smad3) Reverse | TGTGTCGCCTTGTAAGTTCC |  |
| Mouse Tgfb1 (Tgfb1) Forward | CTGCTGACCCCCACTGATAC | NM_011577.2 |
| Mouse Tgfb1 (Tgfb1) Reverse | GGGGCTGATCCCGTTGATT |  |
| Mouse Timp-1 (Timp1) Forward | GAGACACACCAGAGCAGATACC | NM_001044384.1 |
| Mouse Timp-1 (Timp1) Reverse | AAGGTGGTCTCGTTGATTTCT |  |
| Mouse Tnf-α (Tnf) Forward | CTGTAGCCCACGTCGTAGCAAA | NM_013693.3 |
| Mouse Tnf-α (Tnf) Reverse | GACGGCAGAGAGGAGGTTGA |  |

|  |  | **Cirrhosis** | |
| --- | --- | --- | --- |
|  | **Controls** | **Compensated** | **Decompensated** |
| **Number** | 20 | 15 | 25 |
| **Age (years)** | 42.24 ± 2.48 | 61.07 ± 3.09 | 54.35 ± 2.18 |
| **Gender** |  |  |  |
| Male (%) | 47.4% | 60.0% | 57.7% |
| Female (%) | 52.6% | 40.0% | 42.3% |
| **Ethnicity** |  |  |  |
| White (%) | 70.0% | 46.7% | 56.0% |
| South Asian (%) | 10.0% | 40.0% | 40.0% |
| Asian (%) | 5.0% | 6.7% | 0.0% |
| Black (%) | 10.0% | 6.7% | 4.0% |
| **Diabetes (%)** | 0.0% | 86.7% | 20.0% |
| **Heart failure (%)** | 0.0% | 0.0% | 0.0% |

**Supplemental Table 2: Patient demographic information**

Data are represented as the mean ± SEM or percentage (%)

**Supplemental Table 3: Relative liver mRNA expression of genes associated with natriuretic peptide, inflammation, fibrosis and tissue remodelling in murine models of liver disease in WT mice.**

|  | **Std chow** | | | |  | | **CDAA** | | | | **P value vs Std chow** | |  | | **CDAA/Std chow** | | | | **P value vs Std chow** | | **P value vs CDAA** | |  | | **CCl_4_** | | | | **P value vs Std chow** | |
| --- | --- | --- | --- | --- | --- | --- | --- | --- | --- | --- | --- | --- | --- | --- | --- | --- | --- | --- | --- | --- | --- | --- | --- | --- | --- | --- | --- | --- | --- | --- |
| *Cnp* | 1 | ± | 0.21 |  | | 0.50 | | ± | 0.06 | *P=0.0268 | |  | | 0.47 | | ± | 0.03 | *P=0.0171 | | P=0.9995 | |  | | 0.75 | | ± | 0.10 | P=0.4638 | |  |
| *Npr-b* | 1 | ± | 0.11 |  | | 0.37 | | ± | 0.04 | ****P<0.0001 | |  | | 0.47 | | ± | 0.02 | ****P<0.0001 | | P=0.6351 | |  | | 0.26 | | ± | 0.01 | ****P<0.0001 | |  |
| *Npr-c* | 1 | ± | 0.05 |  | | 1.79 | | ± | 0.13 | *P=0.0108 | |  | | 1.50 | | ± | 0.24 | P=0.1638 | | P=0.6603 | |  | | 1.85 | | ± | 0.17 | **P=0.0057 | |  |
| *Il-1*β | 1 | ± | 0.16 |  | | 4.24 | | ± | 0.11 | ****P<0.0001 | |  | | 1.94 | | ± | 0.12 | ***P=0.0004 | | ****P<0.0001 | |  | | 2.00 | | ± | 0.17 | ***P=0.0002 | |  |
| *Il-6* | 1 | ± | 0.06 |  | | 4.06 | | ± | 0.33 | ****P<0.0001 | |  | | 2.23 | | ± | 0.24 | *P=0.0462 | | **P=0.0019 | |  | | 1.68 | | ± | 0.49 | P=0.4713 | |  |
| *Tnf-*α | 1 | ± | 0.07 |  | | 13.60 | | ± | 0.82 | ****P<0.0001 | |  | | 7.30 | | ± | 0.87 | ****P<0.0001 | | ****P<0.0001 | |  | | 1.34 | | ± | 0.09 | P=0.9907 | |  |
| *Il-10* | 1 | ± | 0.16 |  | | 1.53 | | ± | 0.32 | P=0.2131 | |  | | 0.42 | | ± | 0.07 | P=0.1510 | | **P=0.0014 | |  | | 1.00 | | ± | 0.10 | P>0.9999 | |  |
| *Tgf-*β | 1 | ± | 0.08 |  | | 3.17 | | ± | 0.12 | ****P<0.0001 | |  | | 1.64 | | ± | 0.05 | ***P=0.0001 | | ****P<0.0001 | |  | | 2.09 | | ± | 0.08 | ****P<0.0001 | |  |
| *Smad2* | 1 | ± | 0.07 |  | | 0.96 | | ± | 0.06 | P=0.9797 | |  | | 1.11 | | ± | 0.03 | P=0.5976 | | P=0.2943 | |  | | 1.33 | | ± | 0.06 | **P=0.0019 | |  |
| *Smad3* | 1 | ± | 0.09 |  | | 1.06 | | ± | 0.07 | P=0.9983 | |  | | 1.48 | | ± | 0.29 | P=0.1803 | | P=0.2876 | |  | | 0.78 | | ± | 0.11 | P=0.8229 | |  |
| *Fibronectin* | 1 | ± | 0.04 |  | | 1.48 | | ± | 0.03 | *P=0.0279 | |  | | 1.58 | | ± | 0.01 | **P=0.0062 | | P=0.9542 | |  | | 2.67 | | ± | 0.22 | ****P<0.0001 | |  |
| *α-SMA* | 1 | ± | 0.08 |  | | 3.15 | | ± | 0.38 | ***P=0.0006 | |  | | 2.56 | | ± | 0.35 | *P=0.0128 | | P=0.6495 | |  | | 4.46 | | ± | 0.43 | ****P<0.0001 | |  |
| *Col1a1* | 1 | ± | 0.10 |  | | 20.51 | | ± | 1.03 | ****P<0.0001 | |  | | 10.17 | | ± | 0.60 | ****P<0.0001 | | ****P<0.0001 | |  | | 6.56 | | ± | 0.78 | ****P<0.0001 | |  |
| *Col1a2* | 1 | ± | 0.07 |  | | 12.88 | | ± | 0.59 | ****P<0.0001 | |  | | 6.37 | | ± | 0.39 | ****P<0.0001 | | ****P<0.0001 | |  | | 5.11 | | ± | 0.59 | ****P<0.0001 | |  |
| *Col3a1* | 1 | ± | 0.12 |  | | 8.42 | | ± | 0.66 | ****P<0.0001 | |  | | 4.83 | | ± | 0.68 | ****P<0.0001 | | 0.0001 | |  | | 2.08 | | ± | 0.27 | P=0.4452 | |  |
| *Col4a1* | 1 | ± | 0.06 |  | | 2.65 | | ± | 0.09 | ****P<0.0001 | |  | | 1.90 | | ± | 0.13 | ****P<0.0001 | | 0.0001 | |  | | 1.73 | | ± | 0.12 | ***P=0.0002 | |  |
| *Timp-1* | 1 | ± | 0.13 |  | | 64.37 | | ± | 6.98 | ****P<0.0001 | |  | | 33.01 | | ± | 2.69 | ****P<0.0001 | | ****P<0.0001 | |  | | 11.24 | | ± | 0.74 | P=0.2392 | |  |
| *Mmp-13* | 1 | ± | 0.10 |  | | 10.43 | | ± | 0.81 | ****P<0.0001 | |  | | 5.34 | | ± | 0.18 | ****P<0.0001 | | ****P<0.0001 | |  | | 3.36 | | ± | 0.59 | *P=0.0130 | |  |
| *Bax* | 1 | ± | 0.06 |  | | 1.64 | | ± | 0.08 | ****P<0.0001 | |  | | 1.48 | | ± | 0.09 | ***P=0.0004 | | P=0.5006 | |  | | 1.30 | | ± | 0.06 | *P=0.0356 | |  |
| *Bcl2* | 1 | ± | 0.15 |  | | 5.79 | | ± | 0.29 | ****P<0.0001 | |  | | 3.82 | | ± | 0.11 | ****P<0.0001 | | ****P<0.0001 | |  | | 1.60 | | ± | 0.13 | P=0.1135 | |  |

Relative liver mRNA expression of natriuretic peptides, and markers of inflammation, fibrosis and tissue remodelling in models of liver disease. Data are represented as the mean ± SEM. *n* = 7. Statistical analysis by 1-way ANOVA with Šídák post hoc test. *P<0.05, **P<0.01, ***P<0.001 and ****P<0.0001 significantly different from Std chow or CDAA, as indicated.

**Supplemental Table 4: Relative liver mRNA expression of genes associated with natriuretic peptide, inflammation, fibrosis and tissue remodelling in CDAA-fed WT, gbCNP^-/-^, NPR-C^-/-^ and exogenous CNP-treated WT mice.**

|  | **WT** | | |  | **gbCNP^-/-^** | | | **P value vs WT** |  | **NPR-C^-/-^** | | | **P value vs WT** |  | **WT + CNP (P)** | | | **P value vs WT** |
| --- | --- | --- | --- | --- | --- | --- | --- | --- | --- | --- | --- | --- | --- | --- | --- | --- | --- | --- |
| *Cnp* | 1 | ± | 0.12 |  | 0.17 | ± | 0.02 | ****P<0.0001 |  | 0.81 | ± | 0.16 | P=0.5839 |  | 0.63 | ± | 0.12 | P=0.0916 |
| *Npr-b* | 1 | ± | 0.10 |  | 0.88 | ± | 0.14 | P=0.9127 |  | 0.90 | ± | 0.11 | P=0.9480 |  | 1.66 | ± | 0.22 | *P=0.0135 |
| *Npr-c* | 1 | ± | 0.07 |  | 0.94 | ± | 0.11 | P=0.9118 |  | 0.00 | ± | 0.00 | ****P<0.0001 |  | 0.69 | ± | 0.02 | *P=0.0315 |
| *Il-1*β | 1 | ± | 0.03 |  | 1.40 | ± | 0.12 | *P=0.0282 |  | 1.86 | ± | 0.15 | ****P<0.0001 |  | 0.82 | ± | 0.09 | P=0.5339 |
| *Il-6* | 1 | ± | 0.08 |  | 2.70 | ± | 0.69 | *P=0.0118 |  | 1.29 | ± | 0.16 | P=0.9394 |  | 0.72 | ± | 0.09 | P=0.9448 |
| *Tnf-*α | 1 | ± | 0.06 |  | 1.48 | ± | 0.11 | **P=0.0065 |  | 1.41 | ± | 0.12 | *P=0.0270 |  | 0.91 | ± | 0.12 | P=0.9071 |
| *Il-10* | 1 | ± | 0.21 |  | 2.99 | ± | 0.52 | ***P=0.0003 |  | 1.10 | ± | 0.10 | P=0.9943 |  | 0.91 | ± | 0.08 | P=0.9965 |
| *Tgf-*β | 1 | ± | 0.04 |  | 1.08 | ± | 0.06 | P=0.8703 |  | 1.00 | ± | 0.13 | P>0.9999 |  | 0.78 | ± | 0.10 | P=0.2142 |
| *Smad2* | 1 | ± | 0.06 |  | 1.13 | ± | 0.05 | P=0.2760 |  | 1.03 | ± | 0.05 | P=0.9767 |  | 1.17 | ± | 0.06 | P=0.0970 |
| *Smad3* | 1 | ± | 0.07 |  | 1.61 | ± | 0.08 | **P=0075 |  | 0.76 | ± | 0.17 | P=0.4959 |  | 0.89 | ± | 0.19 | P=0.9159 |
| *Fibronectin* | 1 | ± | 0.02 |  | 1.11 | ± | 0.02 | P=0.7362 |  | 0.82 | ± | 0.11 | P=0.3666 |  | 0.63 | ± | 0.14 | *P=0.0144 |
| *α-SMA* | 1 | ± | 0.14 |  | 3.29 | ± | 0.61 | ***P=0.0008 |  | 1.55 | ± | 0.22 | P=0.6978 |  | 1.12 | ± | 0.32 | P=0.9948 |
| *Col1a1* | 1 | ± | 0.05 |  | 1.59 | ± | 0.15 | ***P=0.0008 |  | 0.84 | ± | 0.07 | P=0.5827 |  | 0.83 | ± | 0.08 | P=0.5794 |
| *Col1a2* | 1 | ± | 0.05 |  | 1.47 | ± | 0.12 | *P=0.0171 |  | 0.84 | ± | 0.13 | P=0.6941 |  | 0.88 | ± | 0.14 | P=0.8417 |
| *Col3a1* | 1 | ± | 0.08 |  | 1.84 | ± | 0.17 | **P=0.0042 |  | 2.62 | ± | 0.23 | ****P<0.0001 |  | 1.04 | ± | 0.19 | P=0.9984 |
| *Col4a1* | 1 | ± | 0.03 |  | 1.22 | ± | 0.11 | P=0.7873 |  | 1.55 | ± | 0.22 | P=0.1554 |  | 1.12 | ± | 0.32 | P=0.9597 |
| *Timp-1* | 1 | ± | 0.11 |  | 1.95 | ± | 0.19 | ***P=0.0005 |  | 1.03 | ± | 0.11 | P=0.9985 |  | 0.91 | ± | 0.18 | P=0.9648 |
| *Mmp-13* | 1 | ± | 0.08 |  | 1.79 | ± | 0.24 | P=0.0643 |  | 1.87 | ± | 0.33 | *P=0.0464 |  | 1.48 | ± | 0.26 | P=0.4218 |
| *Bax* | 1 | ± | 0.05 |  | 1.01 | ± | 0.07 | P>0.9999 |  | 1.88 | ± | 0.23 | ***P=0.0001 |  | 1.48 | ± | 0.09 | *P=0.0331 |
| *Bcl2* | 1 | ± | 0.05 |  | 1.00 | ± | 0.07 | P>0.9999 |  | 0.98 | ± | 0.14 | P=0.9973 |  | 0.78 | ± | 0.06 | P=0.2190 |

Relative liver mRNA expression of natriuretic peptides, and markers of inflammation, fibrosis and tissue remodelling in CDAA fed animals. Data are represented as the mean ± SEM. *n* = 6-7. Statistical analysis by 1-way ANOVA with Šídák post hoc test. *P<0.05, **P<0.01, ***P<0.001 and ****P<0.0001 significantly different from WT. Note: WT data in this Table are normalised expression levels from Supplementary Table 3.

**Supplemental Table 5: Relative liver mRNA expression of genes associated with natriuretic peptide, inflammation, fibrosis and tissue remodelling in WT and gbCNP^-/-^ mice treated with CCl_4_.**

|  | **WT** | | | **gbCNP^-/-^** | | | **P value** |
| --- | --- | --- | --- | --- | --- | --- | --- |
| *Cnp* | 1 | ± | 0.13 | 0.21 | ± | 0.02 | ****P<0.0001 |
| *Npr-b* | 1 | ± | 0.05 | 0.94 | ± | 0.15 | P=0.7028 |
| *Npr-c* | 1 | ± | 0.09 | 1.00 | ± | 0.07 | P=0.9761 |
| *Il-1*β | 1 | ± | 0.09 | 1.21 | ± | 0.19 | P=0.3423 |
| *Il-6* | 1 | ± | 0.29 | 2.95 | ± | 0.69 | *P=0.0228 |
| *Tnf-*α | 1 | ± | 0.07 | 2.00 | ± | 0.28 | **P=0.0047 |
| *Il-10* | 1 | ± | 0.10 | 2.01 | ± | 0.25 | **P=0.0026 |
| *Tgf-*β | 1 | ± | 0.04 | 1.12 | ± | 0.05 | P=0.0893 |
| *Smad2* | 1 | ± | 0.05 | 0.90 | ± | 0.03 | P=0.1208 |
| *Smad3* | 1 | ± | 0.14 | 1.84 | ± | 0.08 | ***P=0.0003 |
| *Fibronectin* | 1 | ± | 0.07 | 1.01 | ± | 0.05 | P=0.8814 |
| *α-SMA* | 1 | ± | 0.10 | 11.95 | ± | 5.26 | P=0.0596 |
| *Col1a1* | 1 | ± | 0.12 | 4.54 | ± | 1.09 | **P=0.0071 |
| *Col1a2* | 1 | ± | 0.12 | 3.41 | ± | 0.65 | **P=0.0033 |
| *Col3a1* | 1 | ± | 0.13 | 4.34 | ± | 0.75 | ***P=0.0009 |
| *Col4a1* | 1 | ± | 0.07 | 1.66 | ± | 0.15 | **P=0.0016 |
| *Timp-1* | 1 | ± | 0.07 | 3.19 | ± | 0.75 | *P=0.0128 |
| *Mmp-13* | 1 | ± | 0.18 | 2.92 | ± | 0.33 | ***P=0.0002 |
| *Bax* | 1 | ± | 0.05 | 0.99 | ± | 0.17 | P=0.9744 |
| *Bcl2* | 1 | ± | 0.08 | 1.49 | ± | 0.10 | **P=0.0023 |

Relative liver mRNA expression of natriuretic peptides, and markers of inflammation, fibrosis and tissue remodelling animals treated with CCl_4_. Data are represented as the mean ± SEM. *n* = 6-7. Statistical analysis by unpaired 2-tailed T-Student test. *P<0.05, **P<0.01 and ***P<0.001 significantly different from WT.

**Supplemental Table 6: Relative liver mRNA expression of genes associated with natriuretic peptides, inflammation, fibrosis and tissue remodelling in CDAA fed animals followed by 2 weeks on chow.**

|  | **WT** | | | | **WT + CNP** | | | | **P value** | |
| --- | --- | --- | --- | --- | --- | --- | --- | --- | --- | --- |
| *Cnp* | 1 | ± | 0.07 | 1.17 | | ± | 0.20 | P=0.4464 | |  |
| *Npr-b* | 1 | ± | 0.04 | 1.29 | | ± | 0.04 | ***P=0.0003 | |  |
| *Npr-c* | 1 | ± | 0.16 | 1.06 | | ± | 0.18 | P=0.7971 | |  |
| *Il-1*β | 1 | ± | 0.06 | 0.76 | | ± | 0.08 | *P=0.0323 | |  |
| *Il-6* | 1 | ± | 0.11 | 0.94 | | ± | 0.15 | P=0.7695 | |  |
| *Tnf-*α | 1 | ± | 0.12 | 0.67 | | ± | 0.07 | *P=0.0337 | |  |
| *Il-10* | 1 | ± | 0.17 | 1.82 | | ± | 0.29 | *P=0.0325 | |  |
| *Tgf-*β | 1 | ± | 0.03 | 0.99 | | ± | 0.07 | P=0.9399 | |  |
| *Smad2* | 1 | ± | 0.03 | 0.78 | | ± | 0.05 | **P=0.0041 | |  |
| *Smad3* | 1 | ± | 0.19 | 0.68 | | ± | 0.08 | P=0.1561 | |  |
| *Fibronectin* | 1 | ± | 0.01 | 0.84 | | ± | 0.04 | **P=0.0026 | |  |
| *α-SMA* | 1 | ± | 0.12 | 0.73 | | ± | 0.17 | P=0.2229 | |  |
| *Col1a1* | 1 | ± | 0.06 | 0.98 | | ± | 0.11 | P=0.8633 | |  |
| *Col1a2* | 1 | ± | 0.06 | 0.87 | | ± | 0.10 | P=0.2723 | |  |
| *Col3a1* | 1 | ± | 0.14 | 0.81 | | ± | 0.13 | P=0.3273 | |  |
| *Col4a1* | 1 | ± | 0.07 | 0.90 | | ± | 0.02 | P=0.195 | |  |
| *Timp-1* | 1 | ± | 0.08 | 0.94 | | ± | 0.08 | P=0.6076 | |  |
| *Mmp-13* | 1 | ± | 0.03 | 1.54 | | ± | 0.20 | *P=0.0195 | |  |
| *Bax* | 1 | ± | 0.06 | 0.87 | | ± | 0.05 | P=0.1225 | |  |
| *Bcl2* | 1 | ± | 0.03 | 0.91 | | ± | 0.08 | P=0.2908 | |  |

Relative liver mRNA expression of natriuretic peptides, and markers of inflammation, fibrosis and tissue remodelling in CDAA fed animals followed by 2 weeks on standard chow. Data are represented as the mean ± SEM. *n* = 6-7. Statistical analysis by unpaired 2-tailed T-Student test. *P<0.05, **P<0.01 and ***P<0.001 significantly different from WT.

**Supplemental Figure 1. Graphical summary of the mouse models used.**

**Supplemental Figure 2. Characterisation of the liver disease models**

Phenotyping of mice fed a CDAA diet for 12 weeks (CDAA), mice fed CDAA diet for 12 weeks followed by 2 weeks of chow (CDAA/Std chow), or treated with CCl_4_, compared to mice fed chow (Std chow) for the same period of time. Representative images of spleen and liver (**A**). Liver (**B**) and spleen (**C**) weight normalised to tibia length. Plasma concentration of alanine aminotransferase (ALT; **D**), aspartate aminotransferase (AST; **E**) and CNP (**F**). Representative images of haematoxylin and eosin (H&E) and picrosirius red (PSR) staining (**G**). Quantitative analyses of liver fat (**H**), cell infiltration (**I**), fibrosis (**J**) and MASLD activity score (NAS) (**K**). Statistical analysis by one-way ANOVA with Šídák *post-hoc* test. Each statistical comparison undertaken has an assigned *P* value (adjusted for multiplicity).

**Supplemental Figure 3. Steatosis and portal vein area**

Representative ultrasound images at baseline and after 12 weeks of a CDAA diet from WT and gbCNP^-/-^ mice (**A**). Representative ultrasound images at baseline and after 4 weeks of CCl_4_ administration from WT and gbCNP^-/-^ mice (**B**)

**Supplemental Figure 4. Vascular reactivity characterisation of models of liver disease**

Vascular reactivity in the portal vein isolated from WT and gbCNP^-/-^ mice after CCl_4_ administration for 4 weeks compared to untreated mice (WT Chow): acetylcholine (ACh; **A**), CNP (**B**), cANF^4-23^ (**C**), phenylephrine (Phe; **D**), U46619 (**E**). Data are represented as mean±SEM. Statistical analysis by two-way ANOVA. Each statistical comparison undertaken has an assigned *P* value (adjusted for multiplicity).

**Supplemental Figure 5. Characterisation of the expression of markers driving liver disease**

Relative mRNA expression of natriuretic peptide (NP) signalling, inflammation, fibrosis, tissue remodelling and apoptosis markers in the liver from mice fed CDAA diet for 12 weeks (CDAA), mice fed CDAA diet for 12 weeks followed by 2 weeks of chow (CDAA/Std chow), or treated with CCl_4_, compared to mice fed chow (Std chow) for the same period of time (**A**). p-SMAD-3 phosphorylation and αSMA protein expression in mice fed CDAA diet for 12 weeks (**B**, **C**). Representative images (**D**) and quantification (**E**) of macrophage infiltration (F4/80 staining). Data are represented as mean±SEM. Statistical analysis by one-way ANOVA with Šídák *post-hoc* test. Each statistical comparison undertaken has an assigned *P* value (adjusted for multiplicity).

**Supplemental Figure 6. Characterisation of the expression of natriuretic peptide receptors in hepatic stellate cells.**

Relative mRNA expression (normalised to housekeeping gene RPL-19) of natriuretic peptide receptor (NPR)-A, NPR-B and NPR-C in isolated, differentiated mouse hepatic stellate cells (HSC). Data are represented as mean±SEM.
